# Discovering riboswitches: the past and the future

**DOI:** 10.1016/j.tibs.2022.08.009

**Published:** 2022-09-20

**Authors:** Kumari Kavita, Ronald R. Breaker

**Affiliations:** 1 Department of Molecular, Cellular and Developmental Biology, Yale University, New Haven, CT 06520-8103, USA; 2 Howard Hughes Medical Institute, Yale University, New Haven, CT 06520-8103, USA; 3 Department of Molecular Biophysics and Biochemistry, Yale University, New Haven, CT 06520-8103, USA

**Keywords:** allosteric ribozyme, aptamer, gene regulation, noncoding RNA, metabolite

## Abstract

Riboswitches are structured noncoding RNA domains used by many bacteria to monitor the concentrations of their target ligands and regulate gene expression accordingly. In the past 20 years, over 55 distinct classes of natural riboswitches have been discovered that selectively sense small molecules or elemental ions, and thousands more are predicted to exist. Evidence suggests that some riboswitches might be direct descendants from the RNA-based sensors and switches that were likely present in ancient organisms before the evolutionary emergence of proteins. Herein, we provide an overview of the current state of riboswitch research, focusing primarily on the discovery of riboswitches, and speculate on the major challenges facing researchers in the field.

## 20 years of riboswitch discovery: previous advances and new opportunities

**Figure 1.**
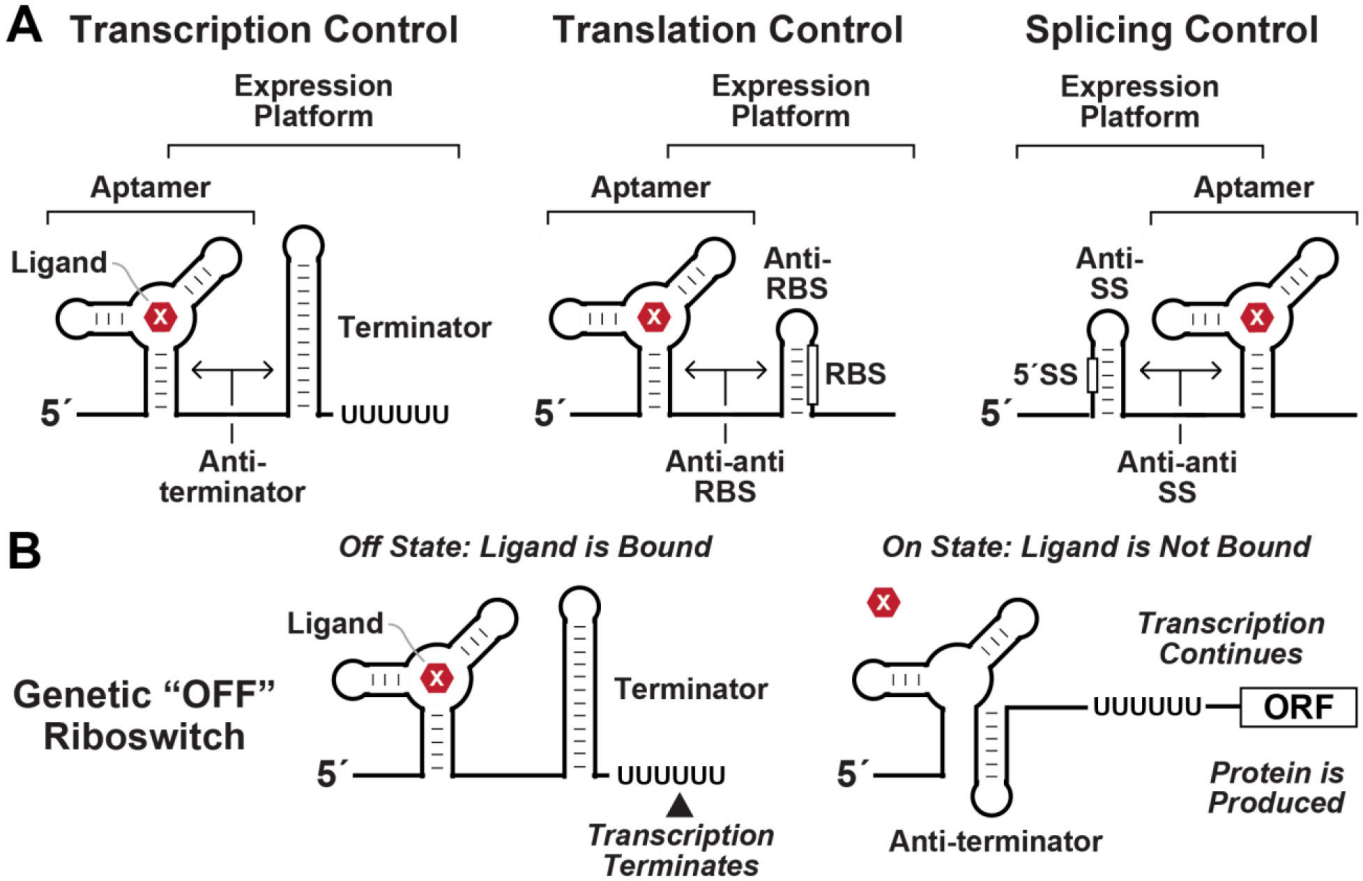
Riboswitch components and mechanisms. (A) Riboswitches typically use partly overlapping aptamer and expression platform domains to regulate transcription termination (left), translation initiation (center), or alternative splicing (right). Rarer mechanisms include transcriptional interference [205] and regulation of mRNA stability [56, 57, 206]. RBS designates the ribosome binding site and SS designates a splice site. Arrows indicate alternative base-pairing that can form in a manner dictated by ligand (X) binding to the notional aptamer structure depicted. (B) Schematic representation of the differences in RNA structure for a genetic “OFF” riboswitch that suppresses gene expression when ligand is bound. If ligand binds the aptamer domain during transcription (left), a folding pathway is favored that forms an intrinsic terminator stem, which triggers transcription termination within a run of U nucleotides. If the ligand is not quickly bound by the aptamer (right), the RNA folds along a different pathway to form the anti-terminator structure. This blocks formation of the terminator stem and promotes transcription of the full mRNA.

Two decades have passed since the first examples of metabolite-binding riboswitches were experimentally validated [1-4]. Each riboswitch usually resides in the 5´ untranslated region (**UTR**) of an mRNA, where it forms at least one ligand-binding **aptamer** domain whose occupancy dictates the folding of an overlapping **expression platform**. Ligand-induced alternative folding of the expression platform is exploited to regulate transcription, translation, or other gene expression process (Figure 1) [5-10]. Because all known riboswitch aptamers bind their target ligands without the need for protein factors, some of these RNAs might be representatives of an ancient sensory and regulatory system that was employed by **RNA World** [11,12] organisms long before proteins emerged in evolution [1,13-15].

**Figure 2.**
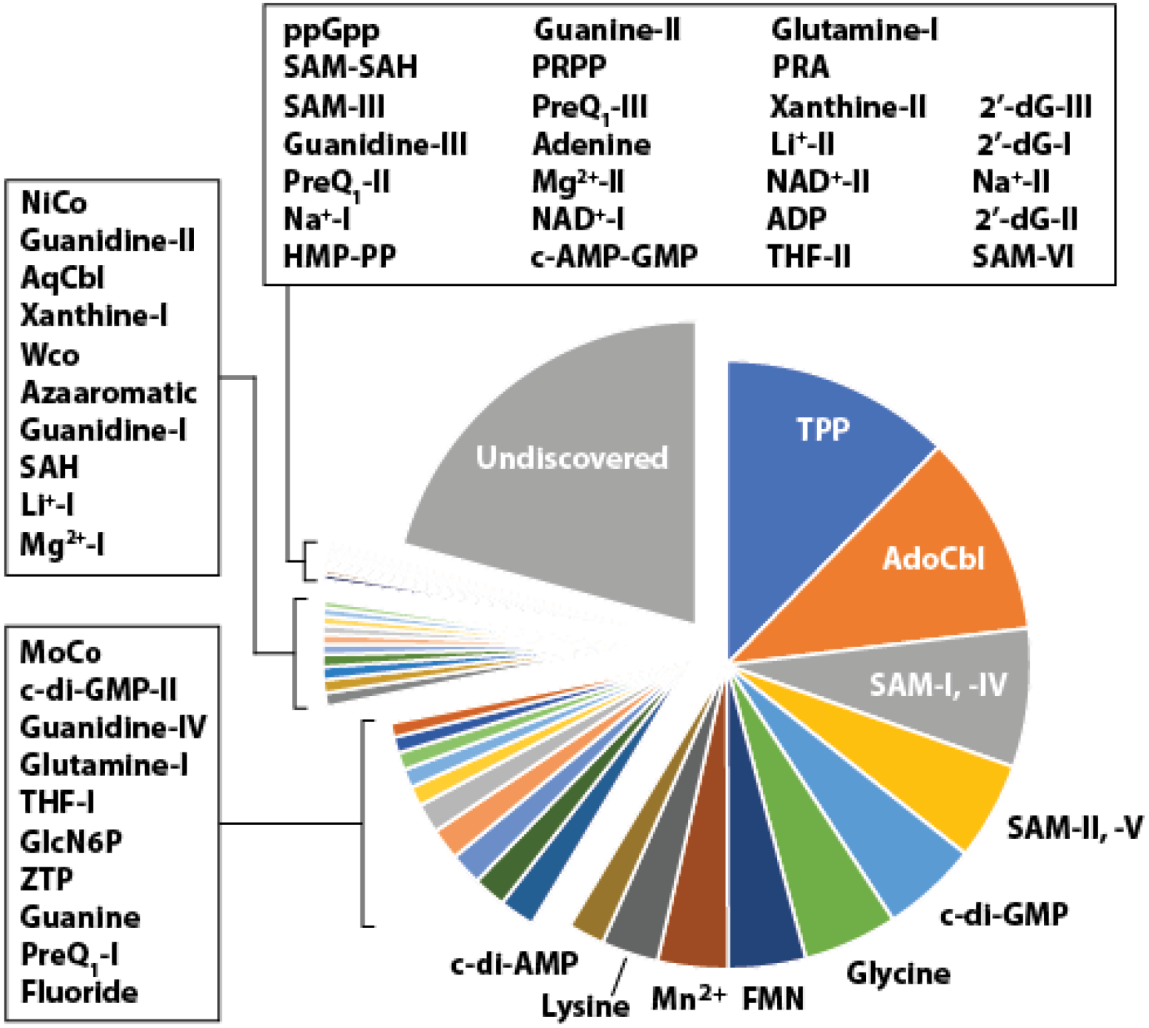
The abundances of experimentally validated riboswitch classes. The abundances of riboswitch classes plotted were obtained from previous publications [7, 16] and are derived from computational searches using databases available at the time of these references. The number of undiscovered riboswitch representatives (~28,000) was estimated using power law projections (Box 2) as described elsewhere [7, 15, 17]. Note that some riboswitch classes are too rare to be visible on the graphic.

The structural features and/or ligand specificity of each aptamer domain are used as a basis for organizing riboswitches into classes (Box 1, Figure I). To date, over 55 distinct riboswitch classes (Figure 2) have been reported that have at least some bioinformatic, genetic, or biochemical data to validate their functions [7,16]. However, this collection likely represents only a tiny fraction of the total number of riboswitch classes that exist in modern organisms; thousands of additional classes are proposed to remain hidden in the genomes of bacteria [7,15,17] (Box 2, Box 3), and we remain hopeful that there are undiscovered examples present in some eukaryotic species.

Although the current list of validated riboswitch classes is small compared to the total predicted, this collection presumably includes the most widespread and abundant examples [7,15]. This assumption is based on our assessment that bioinformatic- and experiment-based riboswitch discovery strategies are more likely to uncover abundant riboswitch classes than rare ones. If true, we can evaluate the functions and mechanisms of the known riboswitch classes with confidence that we are examining the subset of riboswitches that bacteria broadly find to be most useful.

The validated riboswitch classes are also likely to showcase ligand binding and gene control characteristics that are widely exploited by those yet to be discovered. Some of the known riboswitches are remarkably simple in both structure and function [18,19], whereas others employ complicated three-dimensional architectures [20,21] to selectively bind their target and regulate gene expression. Riboswitches often work alone to form very simple ligand-responsive genetic switches, but occasionally they reside in tandem to form sophisticated **natural Boolean logic gates** [22-25]. These examples provide an intriguing preview of the larger diversity of riboswitch structures and functions that await discovery.

Judging by the riboswitch finds over the last two decades, we believe it is worth the time and resources required to further explore these types of noncoding RNA domains. As additional classes are discovered, much more is likely to be learned about the full capabilities of RNA, both as chemical sensors for diverse ligands and as genetic switches. These attributes could have been greatly exploited by early forms of life during the RNA World [14-16]. Furthermore, by examining the genes associated with riboswitches, we can uncover novel connections between ligands and their metabolic or signaling pathways [26,27], thereby revealing the functions of poorly understood proteins or the existence of unusual biological processes [28,29].

A decade after the first metabolite-sensing riboswitch validation studies were reported [1-4], two publications [30,31] provided research progress updates and noted some of the prospects and challenges for riboswitch researchers. In the following sections, we recount some of the recent highlights of riboswitch research since these ten-year assessments were published. In addition, we revisit some of the major topics that remain relevant to the current state of the field, paying special attention to notable research challenges and describing prospects and technical hurdles facing riboswitch researchers in the coming years. In this discussion we do not feature T-box RNAs or RNA thermometers, which are similar types of RNA switches that sense transfer RNAs (tRNAs) or temperature changes, respectively.

## How many riboswitch classes exist in present bacterial species?

Before we discuss details of the known riboswitch classes and the efforts to discover more, it is important to consider how the current state of knowledge compares with what remains unknown. If only a few classes remain to be discovered, then perhaps researchers should move on to new discovery challenges. However, if there indeed are thousands of undiscovered classes, then it seems important to devise approaches that can rapidly shed light on this hidden world of molecular sensors and switches. For the reasons noted below, we believe there are compelling reasons to continue or even increase efforts to discover additional riboswitches and to establish their mechanistic and biological functions.

When estimating the total number of distinct riboswitch classes in modern cells, one must first consider how these RNAs are being classified (Box 1). When making a list of experimentally validated riboswitch classes, we chose to include only those RNAs that form ligand binding pockets for small molecules or elemental ions. As noted above, we exclude other types of RNAs that bind protein factors [32,33], RNA molecules such as tRNAs [34] or small RNAs (sRNAs) [35], and **RNA thermometers** that evaluate temperatures [36]. The sequence and structural features of expression platforms tend to vary considerably and thus are not used to define a riboswitch class. Using these organizational guidelines, we assess that there are at least 56 riboswitch classes that have strong bioinformatic, biochemical and/or genetic evidence for riboswitch function (Figure 2) [7,16].

However, as described later, some riboswitches have resisted researchers’ attempts to classify them, which means that the current rank-order list of validated riboswitch classes is imperfect. Furthermore, it seems certain that additional riboswitches will be found that call into question the above-defined boundaries between ‘classes’ and ‘types’. For example, a series of variants of the long-standing riboswitch candidate called the *ykkC* motif [37] were initially labeled as different ‘types’, but they have since been proven to function as distinct classes that each sense a different ligand [24,29,38,39]. These nomenclature inconsistencies emerge because the classifications are made with incomplete information, and therefore are changed only when the candidates (sometimes hidden as variants of other riboswitch classes) are experimentally validated. These problems will conspire to frustrate the efforts of those seeking to keep a perfect account of riboswitch classes.

Even with these challenges, we believe the current imperfect methods for counting and classifying riboswitches can yield important observations and predictions [14-17,40-43]. Of paramount interest is the task of predicting how many riboswitch classes remain to be discovered in extant species. Given current technologies, it is not possible to definitively establish the total number of riboswitch classes present in modern bacterial species. Simply put, it is simply impractical to gather all species and employ genetic, biochemical or bioinformatic approaches to make an accurate determination. Instead, a method of extrapolation (Box 2) has been used, wherein the abundances of known riboswitch classes are analyzed to predict how many riboswitch classes might exist in the current bacterial genomic sequence databases (Figure II). We estimate that thousands of additional riboswitch classes are likely to be present just among the bacterial species whose genomes have been sequenced [7,15,17].

## The known riboswitch landscape

Although only a tiny sampling of the full diversity of natural riboswitch classes is likely known, several important matters can be evaluated by examining this incomplete list. For example, the expression platforms associated with known riboswitch classes already reveal what mechanisms will commonly be employed by most other riboswitches to regulate genes (Figure 1), as discussed in detail elsewhere [6,9,15,44]. Furthermore, a survey of the ligands sensed by the most common riboswitches help reveal what pathways and processes are critical for cells to regulate [16]. However, the distributions of riboswitches in modern cells are likely shaped by many evolutionary factors, thus making it difficult to draw definitive conclusions based on riboswitch abundance and ligand specificities. Regardless, these considerations help address an intriguing question: do riboswitches represent an ancient system for biochemical sensing and regulation that has its origin in the RNA World?

**Figure 3.**
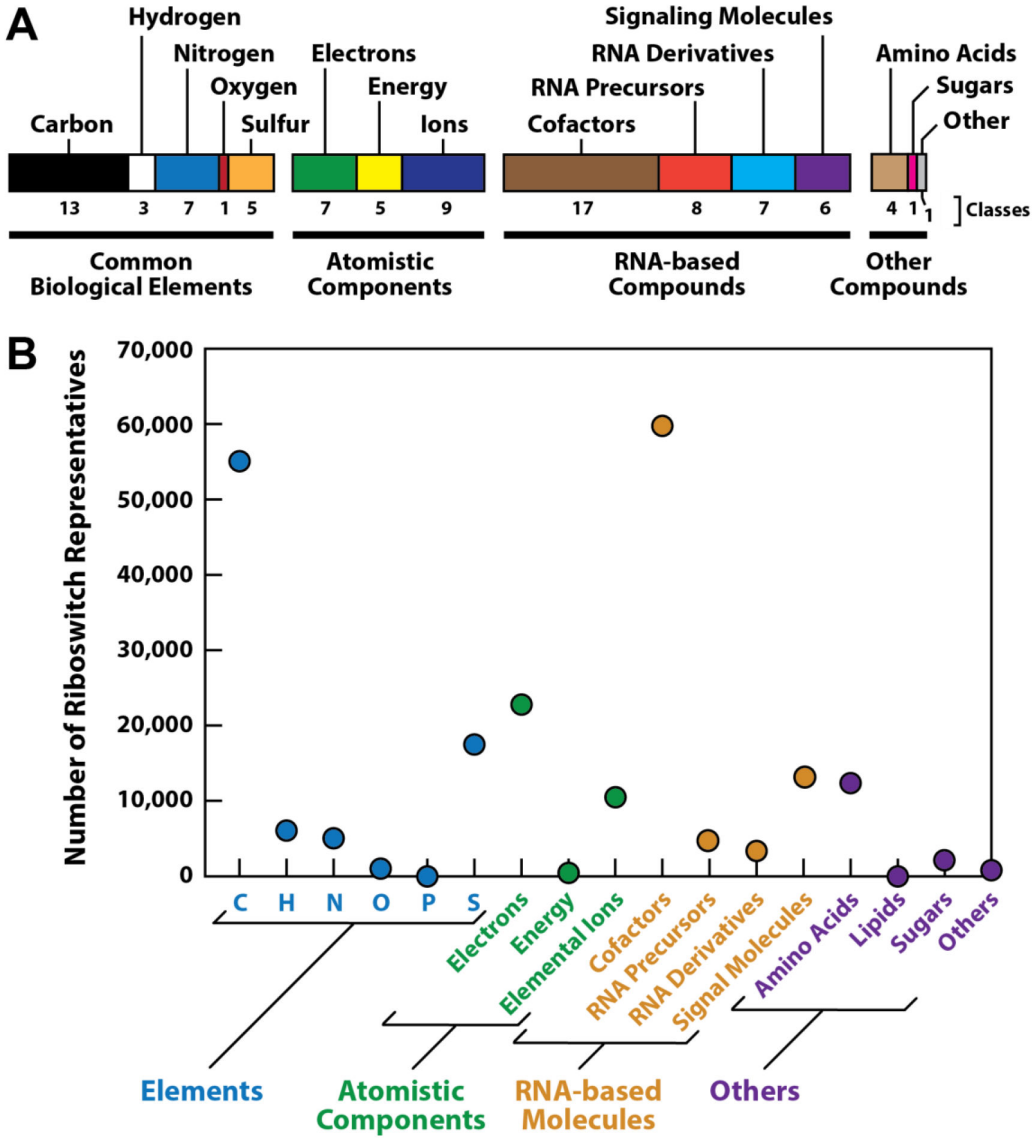
The ligands sensed by the known classes of bacterial riboswitches. (A) Riboswitch classes are assigned to one or more groups based on the roles these ligands serve. The four major categories were chosen to highlight trends relevant to fundamental and ancient biochemical processes. The graphic was adapted from a previous publication [16]. (B) Plot of the number of riboswitch representatives associated with the group assignments as depicted in A. The numbers of representatives for each class were presented elsewhere [16].

Regarding this question, the distribution of ligands sensed by the validated riboswitch classes (Figure 2) provides some intriguing clues. Based on the ligand sensed, each riboswitch class can be organized into one or more broad categories that reflect its larger role in cellular regulation (Figure 3). As an example, THF-I riboswitches [45] are placed into both the ‘Carbon’ (or ‘C’) and the ‘Cofactors’ groups because the tetrahydrofolate (THF) ligand is both a coenzyme and a major contributor to carbon management in cells. This analysis reveals that ‘RNA-based Compounds’ is the most populated of the major ligand categories listed, which is a trend that has previously been noted [7,15]. Riboswitch classes also heavily populate the ‘Common Biological Elements’ and ‘Atomistic Components’ categories, whereas the remaining ‘Other Compounds’ category is only sparsely represented. Bacteria likewise make extensive use of riboswitches to sense many ligands useful for monitoring the status of fundamental biochemical processes such as the homeostasis or manipulation of biologically relevant elements (particularly C, H, N and S), the management of high-energy electrons, and the monitoring of some elemental ions (Box 4).

Consistent with the ‘riboswitches are ancient’ hypothesis is the observation that numerous riboswitch classes have been discovered that sense RNA-like compounds (Figure 3). Several abundant riboswitch classes sense enzyme cofactors that are key mediators of reactions involving the biological elements such as carbon (*e.g.*, TPP, AdoCbl, SAM, or THF), or electrons in the form of hydride units (*e.g.*, NAD^+^ and FMN). These and other enzyme cofactors have been proposed to have emerged in an RNA World [11,12] Therefore, it might be expected that early forms of riboswitches would have been employed by primitive organisms during this era to monitor the concentrations of coenzymes used by enzymes made of RNA.

Also notable are riboswitch classes that sense the building blocks of RNA. For example, a riboswitch class for the nucleotide biosynthetic precursor phosphoribosyl pyrophosphate (PRPP) has been discovered [24,46,47]. PRPP is essential for the de novo biosynthesis of purine and pyrimidine nucleotides and is the source of activated ribose for the recycling of nucleobases. Thus, modern PRPP riboswitches might be descendants of PRPP-binding aptamers or **ribozymes** that also selectively bound this fundamental building block of RNA [41]. Regardless, these and related findings add to the list of fundamental nucleotides, nucleobases and their derivatives known to be sensed by riboswitches [7,16,48,49].

## Riboswitch “blind spots” for ligand sensing

It is interesting to consider the implications of the list of known ligands sensed by riboswitches, but perhaps just as intriguing are the compounds absent from the list. A decade ago, it seemed surprising that there were some fundamental biomolecules that lacked a validated riboswitch class [30]. These included various nucleotide-like compounds such as the enzyme cofactors nicotinamide adenine dinucleotide (NAD^+^) and coenzyme A (CoA), as well as the universal energy currency of all cells, ATP. Over the last ten years, there has been some progress in shortening this list of conspicuously missing riboswitch classes. A detailed discussion of the ligands sensed by experimentally validated riboswitch classes is presented elsewhere [16] and, below, we discuss only a few highlights.

Most notably, two distinct riboswitch classes [50,51] have been discovered that are always associated with genes related to NAD^+^ biosynthesis. One class, called NAD^+^-I, appears to use two similar aptamers to recognize NAD^+^, wherein the second aptamer overlaps a ribosome binding site to suppress gene expression when ligand is bound [50]. Atomistic models established using x-ray crystallographic data [52,53] confirm biochemical evidence that the first aptamer recognizes only the ADP portion of NAD^+^, whereas the second aptamer is predicted [50] to bind the nicotinamide riboside moiety to complete the molecular recognition challenge. However, it has been proposed [53] that the second aptamer also naturally binds ADP as part of an unusual mechanism to regulate NAD^+^ that does not actually involve direct contact with the nicotinamide moiety. We think it is more likely that the second aptamer will prove to bind the nicotinamide moiety tightly and selectively, rather than use a mechanism to measure the levels of NAD^+^ without making physical contact with the chemically unique part of the coenzyme. No such controversy is likely to occur for the second NAD^+^ riboswitch class, called NAD^+^-II [51], which appears to selectively recognize the oxidized form of the nicotinamide ring using an aptamer with a single saturable binding site.

Currently, there are no validated riboswitch classes for CoA or any of its acylated derivatives, such as acetyl-CoA or succinyl-CoA. One possibility is that riboswitches exist for CoA derivatives but, given that acylated versions varying greatly in fatty acyl chain length exist in cells, there is no single common riboswitch class that monitors the CoA pool. Unless the riboswitch is common, it will be difficult to discover by bioinformatic, genetic, or biochemical search strategies. Alternatively, if many of the common riboswitch classes are direct descendants of ancient RNA devices it is possible that RNA World organisms simply had no need for CoA aptamers. It has been proposed that fatty acid (and therefore phospholipid) metabolism emerged late in the evolutionary progression from the RNA World to today’s organisms [45]. If true, then perhaps CoA riboswitches are rare for this reason alone.

Several other fundamental compounds are also missing from the growing list of riboswitch ligands [16], of which some are consistent with the ‘fatty acids late’ evolutionary argument noted above. These include biotin, coenzyme Q (CoQ), and any compounds containing fatty acid moieties such as phospholipids. Biotin is a coenzyme involved in promoting carboxylation reactions that are critical for the biosynthesis of fatty acid chains [54]. CoQ (either menaquinone, plastoquinone, or their derivatives) is a carrier of high-energy electrons that is localized to the hydrophobic center of lipid bilayers and that is an integral part of the electron transport chain of oxidative phosphorylation [55]. Thus, if ancient organisms lacked fatty acid compounds, perhaps they also had little need to sense the coenzymes that are related to the biosynthesis and utilization of these structures.

Other unexpected gaps in riboswitch sensing also exist, and perhaps most noteworthy are several observations listed below. First, there are other near universal coenzymes, namely pyridoxal phosphate, heme, and lipoic acid, that currently lack validated riboswitch classes [16]. Second, although there are many riboswitch classes that sense ligands carrying phosphate groups, no riboswitch classes are known to bind a ligand representing the phosphorus status of the cell, and no riboswitch classes regulate phosphorus homeostasis genes as their primary function. Third, some riboswitches sense ligands that carry a modified ribose moiety, but there are no riboswitch classes that sense an unmodified sugar molecule to regulate carbohydrate metabolism. The closest riboswitch for this latter purpose is the ***glmS* ribozyme** class [56-58], which senses the modified sugar glucosamine-6-phosphate and regulates genes relevant to the production of this modified sugar. If gaps in riboswitch sensing persist in these major areas, then perhaps evolutionary or biochemical reasons for these absences will need to be considered. For example, pyridoxal phosphate is not an RNA-derived coenzyme and thus might not be of ancient origin [45]. Similar arguments might be made for other compounds absent from the list of riboswitch ligands.

Ligands relevant to oxygen management are also notably scarce. Only molybdenum cofactor (MoCo) is counted as a riboswitch ligand whose primary function is relevant to this important task. The predominant role for MoCo-dependent enzymes is to promote oxygen transfer reactions involving redox processes [59]. Perhaps there is little need for most organisms to monitor ligands relevant to metabolic reactions involving oxygen because of its striking abundance, for example in the form of H_2_O and CO_2_. Given that water (~55 molar) is the primary solvent for biological systems, there is an ample supply of hydroxyl groups that can provide an oxyanion nucleophile for hydrolysis or hydroxylation reactions. The removal of an oxygen atom during dehydration reactions produces a water molecule that simply adds to the surrounding solvent.

Although most natural folate derivatives are considered members of a carbon management system, one derivative, 10-formyl-THF (10f-THF) functions as a carrier of an incompletely oxygenated carbon unit. A shortage of this enzyme cofactor leads to the accumulation of the purine biosynthetic intermediate AICAR, which can be further phosphorylated to form the bacterial **alarmone** called ZTP [60]. ZTP is sensed by a riboswitch class that activates the expression of genes involved in 10f-THF biosynthesis [61]. Thus, riboswitches for THF and ZTP also indirectly participate in oxygen management.

The reasons provided above might, at least in part, account for the scarcity of riboswitches directly related to oxygen management. There is a notable absence of riboswitches that either directly bind diatomic oxygen or that can coordinate with this molecule. O_2_ has become a necessity as a recipient of electrons in aerobic organisms that derive energy from reduced electron carriers (*e.g.*, from NADH and FADH_2_) via oxidative phosphorylation. However, conspicuously absent on the list of validated riboswitches classes are those for diatomic oxygen carriers such as heme molecules. Perhaps these are scarce because RNA World organisms might have thrived in an era without atmospheric molecular oxygen [62,63], obviating the need for such RNA sensors during this period and resulting, accordingly, in a dearth of these riboswitch relics in modern organisms.

## Riboswitch structures

Riboswitch research has benefited greatly from the advanced state of RNA structural biology and biophysics. It once seemed possible that the pace of novel riboswitch discoveries would easily be greater than the speed at which atomistic models of their aptamers could be solved by the application of biophysical approaches such as x-ray crystallography or NMR. However, structural models are currently available for nearly all the natural aptamers bound to their ligands [7,64-67]. Indeed, in recent years, X-ray crystallographic structure models for aptamers tend to appear within a few weeks or months of the first report of the existence of the novel riboswitch class [52,68-70], or sometimes even before [71]. Such models provide deep understandings of how RNAs using only the four common types of nucleotides can form diverse, highly selective ligand binding pockets. The speed at which these structural models are established also reduces the need for detailed biochemical analyses, such as complete **structure-activity relationship (SAR) analyses**. Instead of conducting months of expensive analog binding assays with each riboswitch aptamer, quality structural models based on x-ray crystallography or NMR data can provide near comprehensive insight into the nature of each newfound ligand binding pocket.

Although the structural models of riboswitch aptamers are immensely valuable, they provide only a static image of a portion of a riboswitch when bound to its ligand. However, riboswitches also carry an expression platform and are likely to dynamically fold as they are being synthesized during transcription. The important kinetic parameters [72-77] of a riboswitch in its natural setting therefore cannot be fully captured by using traditional structural biology techniques. Fortunately, single-molecule biophysics techniques also have been applied to establish the precise mechanisms by which ligands modulate the fine structures of riboswitch aptamers and expression platforms [*e.g.*, 76,78-82]. These studies can reveal both the pathways and the kinetics of riboswitch folding as they are synthesized and reveal how each base-pair interaction is influenced by the presence of the target ligand or other factors. The techniques of single-molecule biophysics applied to riboswitches have been recently reviewed [83-85] and therefore we will not recount the advances here.

Single-molecule biophysics studies can be complemented by various **RNA-seq** technologies, where the effects of mutations or the status of individual riboswitches *in vitro* [86,87] or eventually *in vivo* [88-90] can be examined in remarkable detail. However, one challenge is that riboswitches even from a single class might employ diverse gene regulation mechanisms and folding pathways that are driven by complicated kinetics- or thermodynamics-driven processes. Therefore, detailed knowledge about a single riboswitch representative from a single organism might not always be informative about the function of a second representative even from the same organism. To make the greatest impact, researchers in the field might need to consider experimental designs or model riboswitch representatives that are likely to reveal broad principles regarding the mechanisms and functions of many riboswitches.

## Where are the eukaryotic riboswitches?

Of all the bacterial riboswitch classes that have been experimentally validated over the last two decades, only one class has convincingly been shown to function naturally in eukaryotic species. TPP riboswitches [2,3], which are the most abundant single class present in bacteria [7], are also relatively common in fungi and plants [91-93]. Studies of several TPP riboswitch representatives in fungi [94-97], algae [98] and plants [99,100] have revealed that they commonly control alternative splicing of pre-mRNA transcripts mediated by spliceosomes [101], but influence gene expression in many different ways.

For example, some fungal TPP riboswitches have been observed to regulate gene expression by retaining or removing an intron located 5′ of the main open reading frame (main **ORF**) [95]. These introns carry one or more upstream open reading frames (uORFs) that suppress main ORF expression by serving as translational decoys. Thus, if TPP is bound to the riboswitch, ribosomes recognize and translate the retained uORFs and ignore the start codon of the main ORF. Similarly, some fungal TPP riboswitches regulate splicing of an intron embedded within the main ORF, where the intron carries a stop codon to cause premature translation termination [95,96]. In algae, TPP ligand binding has been shown to cause intron retention within the main ORF that also carries a stop codon [98]. In plants, TPP binding to some riboswitches causes removal of an intron in the 3′ UTR [99]. This TPP-induced splicing also removes a polyadenylation site, causing a reduction in mRNA stability and suppression of protein synthesis.

Because many eukaryotic species extensively employ alternative RNA splicing [102,103], there should be abundant opportunities for additional riboswitch classes to regulate gene expression via this same general mechanism. Surprisingly, there have been no convincing demonstrations of additional eukaryotic riboswitch classes, despite some intriguing claims. For example, *in vitro* selection for RNA aptamers beginning with pools transcribed from natural genomic DNA sequences from eukaryotes was used to identify numerous RNAs that bind adenosine [104,105], GTP [106,107], or folic acid [108]. An *in vivo* structure probing method also was used to identify putative eukaryotic aptamers for the coenzyme FMN [109]. However, these findings await the publication of convincing evidence that these structures are used by cells as natural aptamers with a relevant biochemical purpose, such as riboswitch function. Also, claims of fungal riboswitches for arginine [110] and spermidine [111] lack sufficient support for riboswitch function, including proper experimental controls, proof of a saturable binding site that can be disrupted by mutation, and evidence for evolutionary conservation among related species.

Even bioinformatic searches have yet to reveal strong candidates for additional eukaryotic riboswitches. As each new bacterial riboswitch is validated, we typically seek homologs in eukaryotes, but usually without success [112]. Also, unbiased searches for novel conserved RNA motifs in plants [113] and fungi [114] have uncovered many novel RNA structures, but none appear to be widespread riboswitch candidates. Despite the current disappointing status of the search for eukaryotic riboswitches, we remain very optimistic that many eukaryotic species, including humans, will be found to make use of riboswitches for metabolites and elemental ions to control various aspects of RNA biology. The transcriptomes of eukaryotes are very large, and there should be many opportunities for ligand-mediated RNA structures to manipulate the important biological processes that include RNA. Introns still appear to be the most promising hunting ground for novel riboswitches, and their ligands might be specialized or more important for regulation in eukaryotic species (*e.g.*, signaling molecules and elemental ions) rather than the fundamental metabolites that are so commonly sensed by bacterial riboswitches [16].

## How (and how not) to find novel riboswitch classes

Both conventional thinking and the **power law** projection for riboswitch abundance [7,15,17] (Box 2) often lead to what we feel are two major misconceptions. The first incorrect interpretation is that the estimated number of undiscovered classes is so large that it simply cannot be true, which causes some researchers to conclude that riboswitch discovery efforts merit no attention. This misconception perhaps leads to inaction on the part of researchers who otherwise might join in the search, but also results in eventual surprise at the ever-growing list of validated riboswitch classes. Even if the estimated number of classes is accepted, it leads to the second incorrect interpretation that there are many novel riboswitch classes hidden in almost every bacterial species. This second problem is far more detrimental because it leads to inefficient choices for strategies to uncover novel classes.

Given that the list of natural riboswitches most probably includes many exceedingly rare classes [7,15,17] (Figure 2, Figure II), it is likely that riboswitch discovery and validation efforts will be relevant long into the future. Therefore, it is important to consider carefully how best to both search for additional classes and how to establish their functions. Each effort to experimentally validate a novel riboswitch is analogous to solving a two-variable equation (Box 3). Solving each ‘riboswitch equation’ requires precise knowledge of both the RNA construct (variable *a*) and the ligand it binds (variable *b*), and the best circumstance is to have high confidence in the answers for these two variables before starting an experimental campaign to prove them. Bioinformatics search algorithms are likely to remain the most effective strategy to identify RNAs that are strong candidates, as well as generate strong clues regarding the precise RNA constructs and the most likely ligand candidates to test. Unfortunately, most other riboswitch discovery strategies proceed without knowledge of either variable, or with knowledge of only one of the two variables. These efforts almost always lead to experimental failures as detailed below for several such methods.

### Genetic searches

Evidence for the existence of riboswitches was first encountered, unknowingly, via the use of genetic analyses conducted by researchers interested in the regulation of specific metabolic pathways. The first reports we can now recognize as hinting at the existence of riboswitches were related to lysine biosynthesis [115,116], and subsequent findings also helped define both the relevant RNA region [117] and the likely ligand [118]. Similar findings were later reported for AdoCbl [119,120], FMN [121,122], guanine [123], MoCo [124,125], and TPP [126], although proof of riboswitch function for each of these examples came years later [1-5,127-129]. A common theme for most of these early genetic studies is that researchers were focused on gene regulation involving a fundamental metabolite, and by chance the underlying riboswitch class turned out to be relatively common. Undoubtedly, this era of riboswitch discovery via single-species genetic analyses has ended. The power law projection (Box 2) predicts the existence of many exceedingly rare riboswitch classes – meaning that on average there currently is less than one undiscovered riboswitch class in each bacterial species. Thus, any genetic screening method that can be applied to identify novel riboswitch classes in an individual organism will frequently fail.

### Genomic SELEX

A search strategy like the **directed evolution** methods used to create novel RNA aptamers [130,131] has been applied to identify natural metabolite binding RNAs. Instead of starting with random-sequence RNA pools, such genomic **SELEX** methods [132] use genomic DNA as a source of templates to produce a diverse population of RNA molecules. The genomic DNA could come from a single species, or from metagenomic samples, with the latter substantially increasing the chance that a novel aptamer will be discovered. Intriguingly, RNA sequences recovered from these studies [104-108] do form binding pockets for the target ligands, but it is not yet certain that they serve biological functions or if they fortuitously exhibit binding. Indeed, the latter seems likely for most of the examples reported, given the predicted rarity of undiscovered riboswitch classes [7,15,17]. For a riboswitch discovery to be made using genomic SELEX, the researchers would need the good fortune of choosing a species or a metagenomic sequence collection that carries at least one riboswitch class, also while choosing to use its matching ligand in the selection process.

### Genome-wide RNA structure probing

Another single-species approach to discovering natural riboswitch aptamers involves the use of *in vivo* or *in vitro* RNA structure probing methods [88-90]. These yield detailed information on the RNA structures as they exist in their natural cellular environments but are unlikely to generate many novel riboswitch class discoveries. Again, the main drawback of these approaches is that the predicted number of novel riboswitch classes per organism studied is simply too small, such that many bacteria have none. Furthermore, structure probing methods can yield signatures of RNA structure switching upon binding of the riboswitch ligand either *in vivo* [133] or *in vitro* [109] but, to successfully establish switching function, the researcher must choose to test the matching ligand for the riboswitch class in the species under examination. Given the low probability of choosing an organism with a novel riboswitch and testing its corresponding ligand from among hundreds or thousands of candidate ligand choices, it is unlikely that researchers can obtain success with this approach at a scale that will be competitive with bioinformatics search methods.

### Transcriptomics searches

Yet another single-species method that has been demonstrated for the discovery of novel noncoding RNA domains involves the analysis of **transcriptomics** data [134,135]. Such methods have proven effective in identifying members of known riboswitch classes, and therefore should also be capable of revealing signatures of novel classes. This strategy takes advantage of the most common riboswitch mechanisms, which lead to transcription termination before the main ORF is transcribed [15,44,136]. Robust expression of the riboswitch domain within the mRNA leader sequence followed by a large reduction in sequence reads within the adjacent ORF is a classic indicator of a riboswitch that is predominantly turning off transcription. However, the same limitations that restrict many other methods again apply here. The probability of examining a species with a novel riboswitch class under conditions that also reveal its ligand is very low.

### Random choice searches

A search strategy wherein the researcher arbitrarily chooses both the RNA construct and the candidate ligand perhaps has the lowest chances for success. Usually, the choice of which gene to study is due to the researcher’s interest in how a particular ligand candidate might regulate gene expression. They then identify a gene that logically might be regulated by the chosen compound. Genetic and biochemical assays ensue, and marginal data is sometimes embraced as evidence for riboswitch function. Again, the problem is that the projected number of riboswitch classes is not in favor of success. Imagine the researcher was fortunate to choose a bacterial species that has a single novel riboswitch class associated with one of its ~4,000 genes. Without additional care, the probability that the researcher has correctly chosen to work on the gene regulated by the riboswitch is 1 in 4,000, and the probability that they have also chosen the correct ligand might be only modestly better. Most validated riboswitch ligands are fundamental metabolites or elemental ions, but some ligands were recognized as biologically relevant only after the candidate riboswitch was identified. Thus, it is possible to choose the right riboswitch construct by chance but not have sufficient knowledge to test its matching ligand.

Given these highly unfavorable probabilities, one should be very skeptical of riboswitch claims based on this search strategy [111,137]. Unfortunately, these claims add intellectual ‘noise’ to the efforts of researchers who seek to understand the biochemical functions and biological roles of riboswitches in general. They are also detrimental because misspent resources on the original experimental validation projects are sometimes followed by investments made by scholars who seek to expand on the false results.

### Bioinformatics searches

Most methods described above could yield novel riboswitch class discoveries, but they are not the high-probability, scalable methods for the discovery of novel riboswitch classes like those needed to substantially advance the field. In contrast, bioinformatics methods [37,138-144] can be applied to entire genomic databases, and they can be indifferent to the riboswitch class or the identities of the ligands sensed. These search algorithms exploit a **comparative sequence analysis** approach [145,146] to reveal novel RNA motifs with conserved sequence and structure features [147-149]. Motifs that also exhibit genomic locations consistent with a regulatory function can provide valuable information to best define the aptamer construct and the candidate ligand to be tested.

## Utility of riboswitches

Natural riboswitches can be exploited in several ways for practical applications, and a full treatment of the latest advances merits a separate review. Herein, we mention only a few major areas in which natural riboswitches can participate in therapeutic and biotechnology advances, and comment on key issues to consider as these technologies mature.

### Riboswitch-targeting antibiotics

Antibacterial compounds that trigger riboswitch function in a manner that is deleterious to the host cell have been developed [150-154]. For example, the application of a ligand analog for a riboswitch that suppresses expression of an essential gene when the analog is bound should cause cell growth inhibition or death. Several features of riboswitches make this an attractive objective. Aptamers form binding pockets for their target ligands, and therefore are predisposed to serve as receptors for drug-like molecules. Each riboswitch ligand could serve as a starting point for analog design to create drug-like derivatives. Various drug screening assays also can be employed to identify artificial ligands [*e.g.*, 155-157], in part by exploiting the natural switching function of the RNAs.

Although some compounds developed to trick riboswitches have been tested in animals [*e.g.*, 158-160], there remain major roadblocks to the practical use of riboswitch-targeting drugs. Only a few riboswitch classes are widespread in pathogenic bacteria [7], which limits opportunities for the development of broad-spectrum antibiotics. Also, it can be relatively simple for mutations to emerge that overcome the effects of some riboswitch-targeting compounds [128,161]. Perhaps the biggest challenge of all is not scientific but is related to the market potential for novel antibiotics [162]. New antibiotics development programs are costly, particularly for clinical trials, whereas the market is fragmented with numerous existing drugs. Unless the financial incentives for antibiotics drug development change, it is likely that riboswitch antibiotics efforts will remain stuck in proof-of-principle stage.

### Engineered riboswitches

Synthetic biologists have been working to create novel aptamers and RNA switches for use as designer gene control devices for more than two decades [163-165]. One objective has been to create RNA molecules that can be used to regulate genes in humans, perhaps delivered by a gene therapy vector. In its simplest form the engineered riboswitch might sense a natural metabolite and regulate gene expression in response to its changing concentrations. Alternatively, expression might be regulated by a synthetic ligand [8]. Protein factors have been considered for this role but presenting a foreign protein in a human might trigger an unwanted immune response, thereby disrupting the regulatory circuit or inducing problematic side effects. An engineered riboswitch is unlikely to cause an analogous immune response, and therefore RNA as a medium for engineered gene control devices has advantages.

Molecular engineers have created various RNA devices that function as switches *in vitro* or *in vivo* [8], but application challenges remain. Aptamers can be created by directed evolution methods [131,166], but these sometimes fail to function in complex cellular conditions. Even if an aptamer exhibits the desired ligand binding specificity and affinity, the aptamer needs to be fused to an mRNA such that expression is regulated by the ligand. A common choice is to fuse an aptamer to a self-cleaving ribozyme to create a ligand-mediated self-destructing RNA, but such arrangements are very rare among natural riboswitches [43]. Perhaps molecular engineers would be better served by exploiting aptamers to regulate alternative splicing [167,168], as is observed with natural eukaryotic riboswitches [95-101] (Box 5).

### Riboswitches as research tools

Many riboswitch classes monitor or regulate such fundamental biochemical pathways that we and others have concluded that they are likely to be of ancient origin [13-15]. Thus, each riboswitch offers researchers a simple mechanism to spy on one or more fundamental biological processes either to monitor normal physiological changes or to identify compounds that perturb cellular processes. Presumably, biosensors derived from many different riboswitches eventually could be created. For example, fluoride riboswitches have been harnessed to serve as components of cell-based biosensors to detect this toxic anion in water samples [169,170].

Riboswitch-reporter fusion constructs also have been utilized to discover novel compounds that perturb biological processes. For example, an *Escherichia coli* strain carrying a plasmid vector expressing a fluoride riboswitch fused to a β-galactosidase reporter gene was used [171] to identify compounds from a chemical library that cause bacterial cells to uptake or retain fluoride – which is toxic at high levels. Compounds like these could be exploited to increase the toxicity of fluoride for use in topical antibacterial agents or in disinfectant formulations. In a similar study, an *E. coli* strain carrying a ZTP riboswitch-reporter fusion construct was used to identify compounds from a chemical library that disrupt the folate cycle [172]. As the list of ligands for natural riboswitches grows, the number of fundamental biological processes that can be likewise monitored also expands.

## Concluding remarks

Natural riboswitches sense a remarkable diversity of ligands and thereby help cells monitor biologically relevant chemicals that are of fundamental importance to all forms of life [7,16]. This list is certain to expand if researchers continue to both establish the functions of **orphan riboswitch** candidates [173] and to identify novel candidates [139-141]. Perhaps some of the prominent riboswitch ‘blind spots’ noted above (Figure 3) will be eliminated, thereby providing further evidence that modern riboswitches robustly contribute to the management of complex metabolic networks, just as their ancient versions likely served.

In addition to finding riboswitch classes that sense more elemental ions (Box 4), that regulate phosphorus metabolism, or that control fatty acid or phospholipid biosynthesis, there are other intriguing possible discoveries that might be made (see Outstanding questions). Several riboswitch classes are known to detect signaling molecules such as c-di-GMP [23,26], c-di-AMP [27], c-AMP-GMP [174,175] and ppGpp [38], or the alarmone ZTP [61]. A variety of other known or possible nucleotide-like signaling molecules are believed to have originated in the RNA World [176,177] and might also have corresponding riboswitches. Signaling molecules such as 3′,5′-cyclic AMP, the putative alarmone Ap4A, and additional forms of cyclic dinucleotides seem like ideal candidate ligands for undiscovered riboswitches [178].

Descriptions of riboswitch mechanism diversity are provided elsewhere [6,9,15,44,101], and it seems likely that additional types remain to be discovered. Surprisingly almost no riboswitch aptamers are known that allosterically regulate the activity of an adjacent ribozyme (Box 5) [43]. As new riboswitch classes continue to trend rarer, there is diminishing hope that there exist large hidden collections of natural **allosteric ribozymes**, which seem almost certain to have once existed in the RNA World. In contrast, there is no shortage of riboswitches arranged in tandem that mimic the operation of Boolean logic gates [22-25]. At least five of the ten possible genetically sensible Boolean logic functions are represented by tandem riboswitch classes [25], and these five include those that result from simply stacking independently functioning representatives. The remaining types of logic functions require a more complex interplay between two aptamer domains, and thus are either rare or perhaps nonexistent in modern cells.

Without enhanced methods, the pace of riboswitch discovery and validation might slow substantially. Traditional laboratory methods are proving inadequate to find more than a few candidates and computational searches will be increasingly frustrated by numerous false positive hits, all due to the rarity of each undiscovered riboswitch class and the abundance of structured noncoding RNAs whose biochemical functions do not involve ligand sensing and gene control. If these problems can be overcome, the next 10 years are likely to reveal many additional surprising structures, functions, and uses of natural riboswitches.

## Box 1: How riboswitches are classified

Each riboswitch class is named after its natural ligand and is delineated based on the structural and functional features of its aptamer domain (Figure I). For example, two aptamers might recognize the same ligand but use distinct RNA architectures (different RNA folds) in their ligand-bound state as judged by their clustering into separate groups either by comparative sequence algorithms or by simple visual inspection. We consider these aptamers to be members of different classes (Figure I, left and middle), as was originally observed for two riboswitch classes that sense *S*-adenosylmethionine (SAM) that are now called SAM-I [178-180] and SAM-II [138]. In contrast, two aptamers might use strikingly similar secondary and tertiary architectures but carry nucleotide differences in or near the ligand binding pocket, resulting in similar binding pockets that recognize different ligands (Figure I, left). The first example of this kind of functional variation was observed for guanine [181] and adenine [182] riboswitches. Given their functional differences, we again consider these to be members of separate riboswitch classes despite their structural similarities.

Finally, in rare instances, two aptamers might recognize the same ligand by forming a near-identical general architecture, but they carry distinct substructural features (Figure I, right). This phenomenon is observed for riboswitches that sense the modified nucleobase 7-aminomethyl-7-deazaguanine (preQ_1_), wherein the preQ_1_-I class [18,183] is represented by RNAs sub-classified as ‘type 1’, ‘type 2’ and ‘type 3’ [184]. Given their near-identical functions and highly similar ligand-binding pockets, these different aptamer ‘types’ were not considered to be separate classes. Intriguingly, a recent study revealed that preQ_1_-I type 1 RNAs form an aptamer structure that binds two preQ_1_ molecules in a stacked configuration [185]. This is an excellent example of the sophistication of even simple riboswitch aptamers, wherein the novel ligand-binding characteristics of preQ_1_-I type 1 RNAs perhaps merit a separate, independent classification from the type 2 and type 3 versions of the original RNA class.

## Box 2. Predicting the total number of riboswitch classes in extant organisms

We observed that the abundances of the most widely distributed riboswitch classes roughly conform to a power law distribution [186], as do many other natural phenomena. Although the riboswitch dataset used for our analyses is incomplete, the abundances of the known riboswitch classes is consistent with this model. For the power law equation *Y* = *mX*^*b*^, the variable *Y* represents the abundance of a riboswitch class (count), *X* represents its place when the classes are ordered according to abundance (rank), *m* is the theoretical number for the most abundant predicted riboswitch class, and *b* is the exponent (slope) for the line resulting when the data is plotted on a log-log plot (Figure II, left). A line with the slope of approximately -1.5 is estimated to best reflect the linear portion of the data points derived from the 56 validated riboswitch classes.

Previously [7], explanations were provided for the observation that the data is non-linear on the power law graph for several of the most common riboswitch classes (*e.g.*, TPP and AdoCbl; top left of the main plot in Figure II), as well as for the rarest classes (bottom right of the main plot). The most common riboswitch classes do not attain the abundances predicted by the power law because cells simply do not need up to five-fold more representatives of TPP or AdoCbl riboswitches to adequately achieve TPP and AdoCbl homeostasis. In other words, the lack of utility for so many riboswitch representatives means that evolutionary forces will limit the abundances of the most common classes. The non-linearity among the rarest classes is almost certainly because it is much more difficult to discover rare riboswitch classes, and therefore we have an incomplete dataset. If true, then new riboswitch class discoveries are predicted to exhibit abundances that will extend the linear portion of the plot.

Intriguingly, there is a large collection of ‘orphan’ riboswitch candidates (see Box 3) that are known to exhibit some characteristics consistent with riboswitch function, but whose ligands remain to be established [139-141,173]. If the longest-standing orphan riboswitch candidates [173] are assumed to represent novel riboswitch classes and are added to the power law graph (Figure II, right), they extend the linear portion of the data without substantively changing the key parameters derived from the power law equation as noted above. Thus, if the trend line depicted on the plot continues, then the true number of riboswitch classes present among the bacterial species whose genomes have already been sequenced (X intercept) is more than two orders of magnitude higher than the number of classes validated to date. Using the same arguments, many more undiscovered classes likely exist among the vast number of bacterial species that have yet to be subjected to genomic DNA sequencing.

## Box 3. Orphan riboswitches

Over the past two decades, bioinformatics methods have been developed [140,141,149] to identify novel classes of structured RNAs – some of which represent promising riboswitch candidates. Many of these RNAs have resisted efforts to identify their target ligands (Figure III), and thus are considered ‘orphan’ riboswitch candidates [37]. At this time, about 100 candidate orphan riboswitch classes await validation [37,139-141,173], each presenting challenges for those who wish to embark on riboswitch validation studies.

Sometimes riboswitches are easily matched with their natural ligand by evaluating the accessory bioinformatics information that can come with the discovery of each new candidate. Specifically, the boundaries of RNA sequence and structure conservation often delimit the minimum sequence required for the aptamer to bind its ligand. Moreover, associated genes can yield valuable clues regarding the ligand identity. When all works well, the two unknowns needed to solve the ‘riboswitch equation’ (*a*, the correct RNA construct; *b*, the ligand identity) can readily be predicted.

Unfortunately, numerous factors can hinder orphan riboswitch validation. Test constructs can be made too short (lacking essential nucleotides) or too long (carry competing structures) and thus fail to exhibit binding or gene control functions. Moreover, RNA constructs of proper length might misfold when prepared outside the environment of their natural host. Particularly frustrating is that gene annotations can be obscure (protein product has an unknown function) or incorrect (wrong function is assigned). Many months of labor can be wasted pursuing flawed hypotheses regarding candidate riboswitch function.

Other factors can also interfere with validation studies despite researchers having gathered solid clues regarding the ligand identity. Riboswitch candidates sometimes are discovered before the ligand is known to science [27], or the ligand is a compound whose biological importance has yet to be established [29]. A comprehensive review of 17 orphan riboswitch classes eventually experimentally validated reveals a total of 12 distinct barriers that were encountered [187, this work]. The list of problems is almost certainly incomplete, and persistence will be needed to make breakthroughs on some of the most difficult orphans.

Future endeavors for riboswitch discovery might rely on using unbiased approaches such as monitoring the activity of riboswitch-reporter fusion constructs in surrogate organisms under various growth conditions predicted to alter intracellular ligand concentrations. Testing chemical libraries, collections of natural compounds, or cell extracts with riboswitch-reporter fusion constructs or prospective RNA aptamer constructs can reveal ligand-triggered RNA functions. However, further purification of candidate ligands from complex mixtures using chromatographic techniques can be labor intensive. Considering that we are in an era in which the most abundant riboswitches have already been validated, and only hard-to-solve or rare riboswitches remain, high throughput and unbiased methods have the edge over the traditional guess-and-test approach. Despite difficulties in ligand assignment for hard-to-solve riboswitch candidates, the reward of gaining scientific knowledge or information on novel areas of biology by establishing the functions of riboswitches is worth the effort.

## Box 4. Riboswitches for elemental ions and metal oxides

Cells must monitor the concentrations of various elemental ions or metal oxides to ensure sufficient concentrations of certain biologically useful elements are present and to guard against the accumulation of toxic levels. Riboswitch classes have already been found that respond to the common cellular ions Mg^2+^ [188,189] and Na^+^ [190,191], as well as other biologically useful ions (Figure IV). Riboswitch classes for adenosylcobalamin [1], molybdenum cofactor [129] and tungsten cofactor [129] bind to compounds that carry a metal center as part of their chemical structures. Representatives of these riboswitch classes sometimes associate with genes whose protein products are involved in metal ion or metal oxide transport and therefore indirectly monitor the abundance of ions such as Co^2+^ or oxides of Mo (molybdate) and W (tungstate). Some have been found to directly sense toxic ions such as lithium (Li^+^) [191] and fluoride (F^−^) [28]. Interestingly, fluoride riboswitches form a selective binding pocket for a tiny negatively charged ion. This is remarkable because RNAs are polyanionic, and yet can partner with Mg^2+^ ions to form a highly selective binding pocket that only binds fluoride [28, 68]. This generates expectations that riboswitches might be found for other negatively charged elemental ions such as chloride.

Numerous other metal ions or metal oxides are reasonable candidates for riboswitches to target (Figure IV). K^+^, Ca^2+^ and Zn^2+^ seem like strong candidate ligands for yet-to-be discovered classes. Lysine riboswitches are known to bind K^+^ as a co-ligand [192], but this riboswitch class seems more likely to be exploited as a regulatory element responsive to lysine rather than K^+^ [127,128]. Also, it is surprising that no proton (H^+^) responsive riboswitches have been reported. The importance of pH homeostasis in cells perhaps limits the potential change in proton concentration that is permitted. In other words, we predict that a proton-sensing riboswitch (if it naturally exists) would need to bind multiple protons in a highly cooperative manner to yield a device that detects exceedingly small changes in pH that would be tolerated by cells.

Experimental validation of metal-ion-responsive riboswitches also has its challenges. The geometries and affinities of coordinated metal ion complexes can be similar, causing ambiguity regarding the natural specificities of riboswitch aptamers [193,194]. This is of particular concern for classes initially demonstrated to respond to Mn^2+^ [195,196] and Ni^2+^ or Co^2+^ [197], but which might naturally include representatives with altered or broader natural ligand specificities [193,194]. Perhaps variants of known metal-ion-binding aptamers exist that broaden the list of ions sensed by riboswitches. Although not depicted in the graphic (Figure IV), there are numerous other metals that might be monitored by cells to prevent their toxic accumulation. Ions or oxides of Cr, Cu, As, Cd, Hg, Pb and many others might be sensed by some species using undiscovered riboswitch classes specialized for these ligands.

## Box 5. Where are the missing allosteric ribozymes?

The first examples of engineered small-molecule-dependent RNA switches were created by fusing aptamer domains to self-cleaving [198-200] or self-ligating ribozymes [201]. The general design strategy is to exploit the energy involved in ligand binding to stabilize RNA structural features that are essential for, or that compete with, substructures important for ribozyme action. This design strategy is exceedingly straightforward, relatively easy to engineer or to manipulate using directed evolution methods, and therefore should be readily accessible via natural evolution. Furthermore, the abundance of natural aptamers [7,16] and various types of self-cleaving [202] and self-splicing [203] ribozymes means that cells have had plenty of opportunities to combine aptamers and ribozymes to create diverse types of cis-acting allosteric ribozyme switches.

Remarkably, there are almost no known examples of allosteric ribozymes in nature [43]. Only one type, formed by the fusion of a c-di-GMP-II aptamer with a group I self-splicing ribozyme, has been reported previously [23]. There are several reasons why direct fusions between aptamers and ribozymes are rare in modern cells. Perhaps foremost among the reasons is that most riboswitches use RNA structures that are far simpler than self-cleaving or self-splicing ribozymes to regulate gene expression. Specifically, many bacterial riboswitches use simple alternative base-pairing interactions to regulate transcription by influencing the folding of an **intrinsic terminator stem** [204] or regulate translation by controlling ribosome access to the Shine-Dalgarno sequence [9]. In eukaryotes, alternative base-pairing is exploited to regulate access to RNA splice sites or polyadenylation signals [101].

Despite the rarity of true allosteric ribozymes, many riboswitches exploit ribozymes in trans to control gene expression (Figure V) [43]. Each riboswitch that regulates translation via mediating access to the Shine-Dalgarno sequence of an mRNA is controlling the instructions read by ribosomes (peptidyltransferase ribozymes). Likewise, riboswitches that control access to splice-site or branch-site sequences regulate spliceosome access to mRNA precursors, which are substrates for these RNA-splicing ribozymes. Thus, many riboswitches operate by exploiting the functions of complex ribozymes encoded elsewhere in their host cell genome.

## Glossary

Alarmone: a natural metabolite produced in response to a stress condition that signals cells to take action to overcome or adapt to the stress.
Allosteric ribozyme: a ribozyme regulated by a ligand via binding to an adjoining aptamer domain.
Aptamer: an RNA sequence that folds to form a binding pocket for a ligand, and in the present context serves as a sensor for a riboswitch.
Comparative sequence analysis: a strategy for assessing important sequence and structural features of RNAs, which yields information on sequence conservation and the presence of base-paired structures, among other characteristics of structured noncoding RNAs.
Directed evolution: methods used to create novel functional biopolymers from random-sequence or mutagenized populations of molecules.
Expression platform: an RNA structure whose folding is affected by the ligand-binding state of an adjoining aptamer, where structural changes affect gene expression.
*glmS* ribozyme: an unusual riboswitch that binds its ligand, glucosamine-6-phosphate, and triggers a self-cleaving reaction that eventually leads to mRNA degradation and suppression of gene expression.
Intrinsic terminator stem: in bacteria, a strong base-paired RNA stem followed by a run of five or more U nucleotides functions to terminate transcription by RNA polymerase, and these structures are commonly exploited as components of expression platforms by riboswitches.
Natural Boolean logic gates: riboswitches can naturally reside in tandem to form genetic devices that mimic the function of two-input Boolean logic gates, where the RNAs sense different ligands and combine their functions to match the truth tables analogous to the gates of electronic logic systems.
ORF: an open reading frame that is read by ribosomes to produce proteins.
Orphan riboswitch: a riboswitch whose matched ligand remains undiscovered or unproven experimentally.
Power law: an equation (*Y* = *mX*^*b*^) that represents the distributions of many natural phenomena in a linear fashion on a log-log plot.
Ribozymes: RNA molecules that form complex structures to catalyze chemical transformations, thereby performing activities like those of enzymes made of proteins.
RNA thermometers: structured RNAs that respond to modest changes in temperature wherein the structural changes alter gene expression.
RNA World: a proposed era in early evolution were RNA molecules served to both store genetic information (genotype) and promote chemical catalysis (phenotype), long before DNA and proteoins emerged in evolution.
RNA-seq: methods used to establish the sequences of large collections of variable-length or variable-sequence RNAs.
SELEX: a general term for directed evolution methods applied to populations of RNA or DNA molecules.
Structure-activity relationship (SAR) analyses: for riboswitches, this is a process wherein subtle chemical changes are made to ligands to determine the important contacts recognized by the riboswitch aptamer.
Transcriptomics: methods to collect and analyze the diversity of RNA sequences in biological samples.
UTR: an untranslated region either at the 5ór 3énd of an mRNA, which are typical locations for riboswitches.
